# Lack of evidence for the presence of Schmallenberg virus in mosquitoes in Germany, 2011

**DOI:** 10.1186/1756-3305-7-402

**Published:** 2014-08-29

**Authors:** Kerstin Wernike, Hanna Jöst, Norbert Becker, Jonas Schmidt-Chanasit, Martin Beer

**Affiliations:** Institute of Diagnostic Virology, Friedrich-Loeffler-Institut, Suedufer 10, 17493 Greifswald, Insel Riems, Germany; Department of Virology, Bernhard Nocht Institute for Tropical Medicine, Bernhard-Nocht-Straße 74, 20359 Hamburg, Germany; German Centre for Infection Research (DZIF), partner site Hamburg-Luebeck-Borstel, Hamburg, Germany; German Mosquito Control Association (KABS), Waldsee, Germany

**Keywords:** Schmallenberg virus, Orthobunyavirus, Arbovirus, Vector, Mosquito, Transmission

## Abstract

**Background:**

In 2011, a novel orthobunyavirus of the Simbu serogroup was discovered near the German-Dutch border and named Schmallenberg virus (SBV). So far, SBV genome has been detected in various field-collected *Culicoides* species; however, other members of the Simbu serogroup are also transmitted by mosquitoes.

**Findings:**

In the present study, approximately 50,000 mosquitoes of various species were collected during summer and early autumn 2011 in Germany. None of them tested positive in an SBV-specific real-time PCR.

**Conclusions:**

The absence of SBV in mosquitoes caught in 2011 in Germany suggests that they play no or only a negligible role in the spread of the disease.

## Findings

### Introduction

Schmallenberg virus (SBV), the first European member of the Simbu serogroup, genus *Orthobunyavirus*, emerged in summer 2011 near the German/Dutch border [1]. Since then, the virus has spread very rapidly over large parts of the continent. Affected adult ruminants show either no or non-specific, mild clinical signs for only a few days, but fetal infection may lead to severe malformation, stillbirth or premature birth [2].

Simbu serogroup viruses have been frequently isolated from *Culicoides* midges, but also from mosquitoes [3, 4]. So far, SBV has been detected in various *Culicoides* species such as *C. obsoletus* s.s., *C. scoticus*, *C. chiopterus*, *C. dewulfii*, *C. pulicaris*, or *C. nubeculosus* collected during summer and early autumn 2011 in Belgium, the Netherlands or Denmark [5–7]. Of head pools from *Culicoides* midges collected in the Netherlands throughout September and early October 2011 2.3% tested positive by real-time RT-PCR [5], and an infection rate of approximately 3.6% was estimated for *Culicoides* caught in the region of Antwerp (Belgium) in September 2011 [6].

However, in hibernating mosquitoes SBV was not detected which suggests that mosquitoes are not important for the persistence of SBV during winter [8]. However, their role in SBV-transmission during the period of high virus circulation is unknown.

### Methods

**Figure 1:**
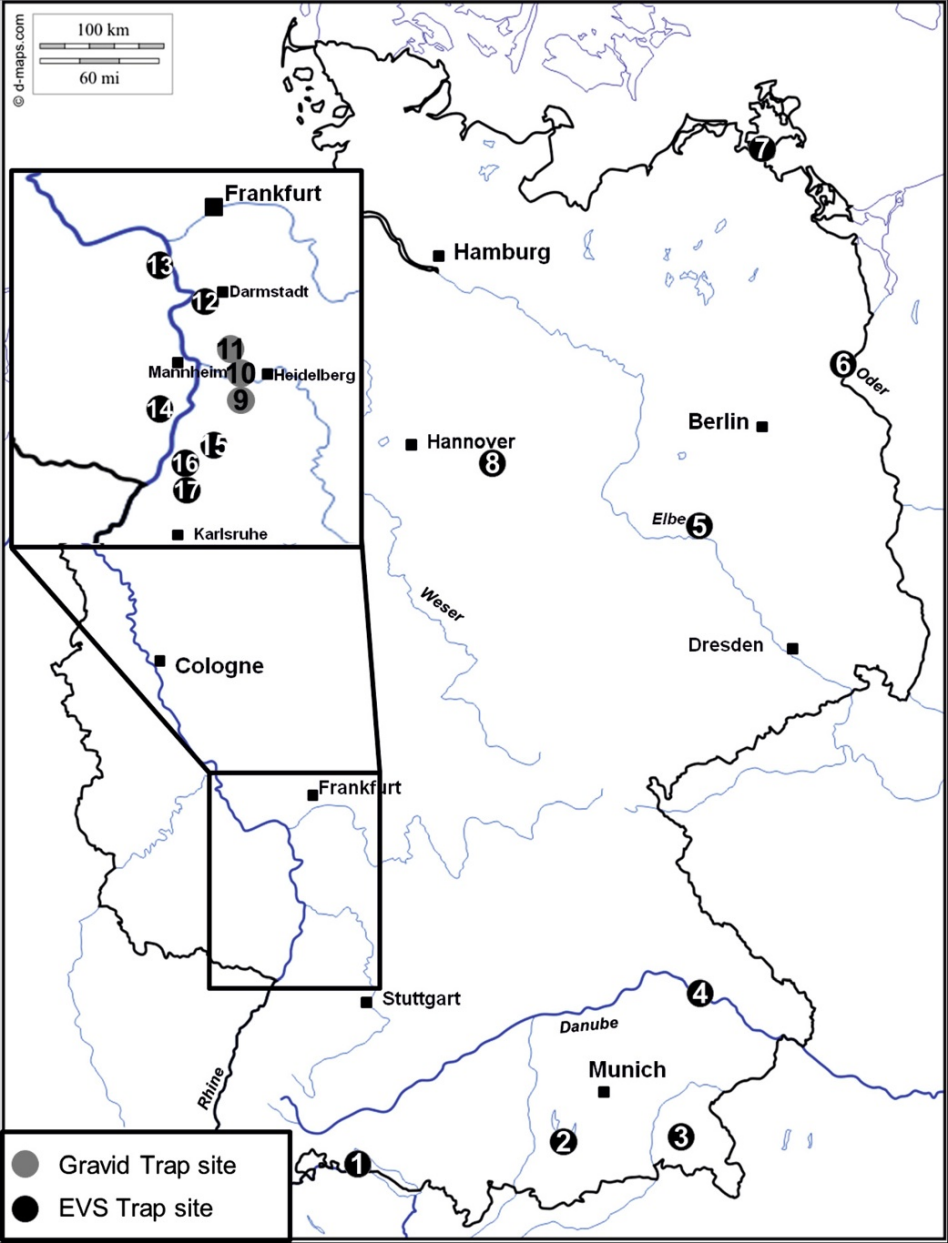
**legend: Location of the trapping sites.**

In the present study, female mosquitoes were collected in summer and early autumn 2011 at 17 sites in Germany (Figure 1). The mosquitoes were either trapped with CO_2_-baited encephalitis vector surveillance (EVS) traps (BioQuip, Compton, CA) or gravid traps (GT) designed according to the CDC gravid trap model 1712 (John W. Hock Company, Gainesville, FL). Collected mosquitoes were deep-frozen transported to the laboratory and subsequently identified on chill tables according to species and sex using morphological characteristics [9]. Mosquitoes were pooled (up to 25 specimens) according to species and trapping site, placed in sterile 2-ml cryovials, and then maintained at −70°C until being tested for virus RNA. The homogenization of mosquitoes was done according to Jöst *et al.* [10]. Total RNA was extracted using the QIAamp viral RNA mini kit (Qiagen, Hilden, Germany) according to manufacturer’s recommendation, and tested by an SBV S-segment specific real-time RT-PCR [11] which has been previously used for SBV-detection in pools of midges (up to 50 midges per pool) [5, 6, 12].

### Results and discussion

Between May and September 2011, a total of 50,708 mosquitoes were collected. The most abundant species trapped were *Culex pipiens/torrentium* (62%) and *Aedes vexans* (24%). The number of individuals and the species are listed in Table 1 individually for each trapping site. Most of the individuals collected in GT are gravid females, which had already taken a blood meal, making them more suitable for arbovirus surveillance. All mosquitoes collected in summer and early autumn 2011 in Germany tested negative in the SBV-specific real-time PCR. During this period, an unidentified disease, which was later identified as an infection with SBV was reported in German and Dutch dairy cattle herds [1]. From August onwards, SBV-specific antibodies were detected in domestic ruminants [13] suggesting a circulation of virus during the trapping period. After the 2011 epizootic, the seroprevalence in cattle reached nearly 100% in the focus of the affected area, and the virus had spread very rapidly over large parts of Europe [14, 15]. SBV was even detected in *Culicoides* midges caught in Denmark in October or in Italy between September and November 2011 (reviewed in [14]). In the German federal state Rhineland-Palatinate, the seroprevalence in cattle was approximately 80% (95% confidence interval (CI) 67.67 - 89.22%) after the 2011 epizootic, and in Baden-Wuerttemberg it was about 32% (95% CI 22.23 - 44.10%) [14], the trapping sites 9 to 17, where more than half of the mosquitoes were collected, are located in the border region of both federal states. Despite this very high prevalence in the ruminant hosts and the thereby presumably considerable virus circulation, none of the mosquitoes collected in the present study tested positive by the SBV-specific real-time RT-PCR. However, approximately one third of the tested mosquitoes were caught in Mecklenburg-Pomerania (trapping site 7), a region with a seroprevalence of only about 2% (95% CI 0.06 – 12.29%) in cattle [14].

**Table 1:** **Trapping sites, dates, and number of mosquitoes per species collected during the study period**

	Location number on map	Trapping date	Number of trappingperiods	Trap type	Culex modestus*	***Culex pipiens/ torrentium***	***Culex territans***	Aedes vexans*	Aedes cinereus*	Aedes rossicus*	Ochlerotatus annulipes*	Ochlerotatus cantans*	Ochlerotatus communis*	Ochlerotatus geniculatus*	Ochlerotatus punctor*	***Ochlerotatus rusticus***	***Ochlerotatus sticticus***	Ochlerotatus caspius*	Ochlerotatus flavescens*	Culiseta annulata*	***Anopheles claviger***	***Anopheles maculipennis***	Anopheles plumbeus*	Mansonia richiardii*	total no of mosquitoes
Alsheim	13	27-28.07.2011	1	EVS	2	8	25	1	1	0	0	0	0	0	0	0	0	0	0	13	0	6	0	0	**56**
Lake Constance, Radolfszell	1	02-03.08.2011	1	EVS	0	71	0	33	22	0	0	0	0	0	0	11	0	0	0	4	0	1	0	1	**143**
Lake Chiemsee	3	03-04.08.2011	1	EVS	0	100	0	265	85	0	0	0	0	0	0	0	226	0	0	1	1	0	0	0	**678**
Drömling	8	18-19.08.2011	1	EVS	0	14	0	9	3	0	0	0	0	0	0	2	0	0	0	1	0	0	0	0	**29**
Elbe, Coswig	5	15-16.08.2011	1	EVS	0	194	0	863	8	0	0	0	0	0	0	0	0	0	1	1	0	2	0	0	**1069**
Greifswald	7	17-18.08.2011	1	EVS	0	11605	0	2839	1629	4	13	433	16	0	225	17	346	79	43	31	43	78	0	0	**17401**
Großsachsen	10	May-September 2011	61	GT	0	5081	0	0	0	0	0	0	0	0	1	0	0	0	0	11	1	0	1	0	**5095**
Haßloch	14	10-11.05.2011	1	EVS	0	9	0	8	11	10	52	255	11	0	21	1702	7	0	0	6	2	9	0	0	**2103**
Heidelberg	9	May-September 2011	41	GT	0	9581	0	0	0	0	0	1	0	0	0	0	0	0	0	7	2	2	0	0	**9593**
Insel Rott	17	26-27.07.2011	1	EVS	0	0	16	137	5	0	0	6	0	0	0	0	4	0	0	3	1	10	0	0	**182**
Isar, Schiltorn	4	04-05.08.2011	1	EVS	0	41	0	402	15	0	0	0	0	0	0	88	13	0	0	4	5	1	0	25	**594**
Kühkopf	12	27-28.07.2011, 10–11.08.2011, 16–17.08.2011, 23–24.08.2011	4	EVS	0	208	0	6237	18	0	1	2	0	0	0	0	0	0	0	23	5	45	2	0	**6541**
Oder, Hohenwutzen	6	16-17.08.2011	1	EVS	0	1003	0	1107	69	6	3	20	0	0	4	6	1	1	4	16	0	55	0	0	**2295**
Osterseen, Iffelsdorf	2	03-04.08.2011	1	EVS	0	41	0	97	380	0	0	1	0	0	8	0	0	0	0	3	1	0	0	2	**533**
Rußheimer Altrhein	16	26-27.07.2011	1	EVS	0	37	0	300	6	0	1	3	0	0	0	0	9	0	0	9	0	27	0	0	**392**
Waghäusel	15	07-08.06.2011, 12–13.07.2011	2	EVS	0	32	6	0	0	19	45	168	0	0	0	2	0	0	0	16	125	20	0	4	**437**
Weinheim	11	May-September 2011	78	GT	0	3546	0	0	0	0	0	0	0	0	0	0	0	0	0	20	0	0	0	1	**3567**
**Total no of mosquitoes**					**2**	**31571**	**47**	**12298**	**2252**	**39**	**115**	**889**	**27**	**0**	**259**	**1828**	**606**	**80**	**48**	**169**	**186**	**256**	**3**	**33**	**50708**

EVS: encephalitis vector surveillance traps; GT: gravid trap; mammophilic species are marked with * according to Becker *et al.* [16].

Total numbers of mosquitos are printed in bold type.

In Australia, Asia or Africa, Simbu viruses can be isolated from local mosquitoes [3, 4]. Since SBV is the first European member of the Simbu serogroup, species potentially involved in transmission in Europe cannot be deduced from closely related viruses. However, several mosquito-borne mammal-associated orthobunyaviruses of other serogroups such as Ťahyňa virus, Inkoo virus (both California serogroup*)* or Batai virus (Bunyamwera group) have been documented in various western European countries [17]. Of these, Ťahyňa virus is most often isolated from *Aedes vexans*, which was the second most common species trapped in the present study, but also from other culicine mosquitoes. The principal vector for Batai virus in Europe are zoophilic mosquitoes such as *Anopheles maculipennis* s.l., *Anopheles claviger*, *Ochlerotatus punctor* and *Ochlerotatus communis*, among others [18]. All of these species were collected in the present study and tested for the presence of SBV.

Despite reported symptoms of the disease in susceptible animals during the trapping period and a high seroprevalence after the first vector season, none of the collected mosquitoes tested positive in the SBV-specific real-time RT-PCR. Considering the detection of viral RNA in biting midges in regions with a much lower seroprevalence in ruminants, in Denmark even before clinical signs were observed or virus was detected in domestic animals [19], mosquitoes most likely play only a negligible, if any, role in SBV transmission.

## Abbreviations

SBV: Schmallenberg virus
EVS: Encephalitis vector surveillance
GT: Gravid trap.

## References

[1] Hoffmann B, Scheuch M, Höper D, Jungblut R, Holsteg M, Schirrmeier H, Eschbaumer M, Goller KV, Wernike K, Fischer M, Breithaupt A, Mettenleiter T, Beer M (2012). Novel orthobunyavirus in cattle, Europe, 2011. Emerg Infect Dis.

[2] Beer M, Conraths FJ, van der Poel WH (2013). 'Schmallenberg virus' - a novel orthobunyavirus emerging in Europe. Epidemiol Infect.

[3] Elliott RM, Blakqori G, Plyusnin A, Elliott RM (2011). Molecular biology of orthobunyaviruses. Bunyaviridae: Molecular and Cellular Biology.

[4] Saeed MF, Li L, Wang H, Weaver SC, Barrett AD (2001). Phylogeny of the Simbu serogroup of the genus Bunyavirus. J Gen Virol.

[5] Elbers AR, Meiswinkel R, van Weezep E, van Oldruitenborgh-Oosterbaan MM S, Kooi EA (2013). Schmallenberg Virus in Culicoides spp. Biting Midges, the Netherlands, 2011. Emerg Infect Dis.

[6] De Regge N, Deblauwe I, De Deken R, Vantieghem P, Madder M, Geysen D, Smeets F, Losson B, van den Berg T, Cay AB (2012). Detection of Schmallenberg virus in different Culicoides spp. by real-time RT-PCR. Transboundary and emerging diseases.

[7] Rasmussen LD, Kristensen B, Kirkeby C, Rasmussen TB, Belsham GJ, Bodker R, Bøtner A (2012). Culicoids as vectors of schmallenberg virus. Emerg Infect Dis.

[8] Scholte EJ, Mars MH, Braks M, DENH W, Ibanez-Justicia A, Koopmans M, Koenraadt JC ADEV, Reusken C (2013). No evidence for the persistence of Schmallenberg virus in overwintering mosquitoes. Med Vet Entomol.

[9] Becker N, Petric D, Zgomba M, Boase C, Dahl C, Lane J, Kaiser A (2003). Mosquitoes and their control.

[10] Jöst H, Bialonski A, Storch V, Gunther S, Becker N, Schmidt-Chanasit J (2010). Isolation and phylogenetic analysis of Sindbis viruses from mosquitoes in Germany. J Clin Microbiol.

[11] Bilk S, Schulze C, Fischer M, Beer M, Hlinak A, Hoffmann B (2012). Organ distribution of Schmallenberg virus RNA in malformed newborns. Vet Microbiol.

[12] Elbers AR, Meiswinkel R, van Weezep E, Kooi EA, van der Poel WH (2014). Schmallenberg Virus in Culicoides Biting Midges in the Netherlands in 2012. Transboundary and emerging diseases.

[13] Veldhuis AM, van Schaik G, Vellema P, Elbers AR, Bouwstra R, van der Heijden HM, Mars MH (2013). Schmallenberg virus epidemic in the Netherlands: Spatiotemporal introduction in 2011 and seroprevalence in ruminants. Prev Vet Med.

[14] Wernike K, Conraths F, Zanella G, Granzow H, Gache K, Schirrmeier H, Valas S, Staubach C, Marianneau P, Kraatz F, Höreth-Böntgen D, Reimann I, Zientara S, Beer M (2014). Schmallenberg virus-Two years of experiences. Prev Vet Med.

[15] EFSA: *"Schmallenberg" virus: analysis of the epidemiological data (May 2013).* EFSA Supporting Publications 2013 EN-3429;http://www.efsa.europa.eu/de/supporting/doc/429e.pdf

[16] Becker N, Kruger A, Kuhn C, Plenge-Bonig A, Thomas SM, Schmidt-Chanasit J, Tannich E (2014). [Mosquitoes as vectors for exotic pathogens in Germany]. Bundesgesundhbl. Gesundheitsforsch. Gesundheitsschutz.

[17] Lundström JO (1999). Mosquito-borne viruses in western Europe: a review. Journal of vector ecology: journal of the Society for Vector Ecology.

[18] Hubalek Z (2008). Mosquito-borne viruses in Europe. Parasitol Res.

[19] Rasmussen LD, Kirkeby C, Bodker R, Kristensen B, Rasmussen TB, Belsham GJ, Bøtner A (2014). Rapid spread of Schmallenberg Virus-infected Biting Midges (Culicoides spp.) across Denmark in 2012. Transboundary and Emerging Diseases.

